# Effectiveness of structured psycho-oncological counseling for relatives of lung cancer patients based on the CALM approach—study protocol of a randomized controlled trial

**DOI:** 10.1186/s13063-024-07954-9

**Published:** 2024-02-10

**Authors:** Julia Dusel, Karin Meng, Hanna Arnold, Antonia Rabe, Elisabeth Jentschke

**Affiliations:** University Hospital Würzburg, Comprehensive Cancer Center Mainfranken, Josef-Schneider Str. 6, Würzburg, D-97080 Germany

**Keywords:** Psycho-oncology, Lung cancer, Family care, Counseling, Oncology, CALM

## Abstract

**Background:**

The high incidence combined with the high lethality and bad prognosis of lung cancer highlight the need for psycho-oncological care for both patients and their relatives. While psychological interventions for relatives might be helpful, further research on the impact of specific interventions is necessary. Therefore, this trial aims to evaluate structured psycho-oncological counseling for relatives of lung cancer patients based on the Managing Cancer And Living Meaningfully (CALM) approach compared to usual care. In addition, we explore the impact of psycho-oncological support of relatives on the patients’ mental health outcomes.

**Methods:**

The study is a single-center, prospective, randomized controlled trial with two measurement time points. Relatives of lung cancer patients and, thus, the patients themselves (i.e., dyads) are randomly allocated to the intervention group (IG) or the control group (CG) regardless of their disease or treatment stage. Relatives in the IG receive structured counseling based on the CALM approach (three to six sessions with psycho-oncologists). The CG receives usual psycho-oncological care. In addition, cancer patients in both study arms can request psycho-oncological support (usual care) as needed, but they will not get a specific intervention. Relatives and patients complete assessments at baseline (T0) and after the intervention/6 weeks (T1). The primary outcome for relatives is anxiety. Relatives’ secondary outcomes include depressive symptoms, distress, supportive care needs, and quality of life. Patients’ outcomes include anxiety, depression, and distress. All outcomes are assessed using self-report validated questionnaires. Intervention effects will be evaluated using analysis of covariance (ANCOVA) adjusting for baseline values. Power calculations reveal the need to enroll 200 subjects to detect an effect of *d* = 0.4.

**Discussion:**

The study will provide evidence for the effectiveness of the CALM intervention in relatives of lung cancer patients. Furthermore, study results will contribute to a better understanding of the effectiveness of a psycho-oncological intervention for highly impaired cancer patients and their relatives. If the CALM intervention positively affects the relatives’ psychosocial outcome, it may be implemented in routine care.

**Trial registration:**

German Clinical Trials Register DRKS00030077. Retrospectively registered on 26 October 2022.

**Supplementary Information:**

The online version contains supplementary material available at 10.1186/s13063-024-07954-9.

## Background and rationale

In Germany, every year, more than 55,000 people are diagnosed with malignant tumors of the lung. The annual incidence of lung cancer ranks second among men and third among women compared to all other types of cancer [1]. Lung cancer ranks first among men (22.8%) and second among women (15.8%) of all cancer deaths in Germany. Despite advances in diagnosis and therapy, the 5-year survival rate is only 22% in women and 17% in men, and less than half of patients survive the first year after diagnosis [2].

Cancer is often accompanied by psychological and social stress or mental disorders [3]. Particularly lung cancer patients experience psychological distress due to low survival rates, physical discomfort associated with the disease and treatment, and poor quality of life [4]. A recent meta-analysis shows that nearly 50% of all lung cancer patients show high levels of psychological distress [5]. Furthermore, studies report a high incidence and prevalence of depression and anxiety [6–8]. The prevalence of a major depressive disorder is 14.4%, correlating with poor quality of life, fatigue, and sleep disturbances [9]. Overall, data on psychological burden highlight the need for psycho-oncological support [3].

Cancer is not only a burden for the patients themselves, but it also negatively impacts their relatives’ psychological and physical well-being [10]. However, family members often neglect their own needs by focusing on the disease of their relatives [11, 12]. Because of late diagnosis, rapid progression, and poor prognosis of bronchial cancer, relatives are particularly vulnerable and report higher levels of distress than do relatives of other cancer patients [13, 14]. Some studies show that the relatives’ emotional burden may be even higher than the patients’ [13, 15–17]. In addition, the physical and psychological health of cancer patients and their relatives are interdependent [18, 19]. Psychological support for family members positively in turn affects the patient’s psychosocial outcomes. Lung cancer patients with higher resilience and social support have lower levels of anxiety and depression [20].

A recent review reported that depression and anxiety disorders and psychological distress are significantly associated with an increased risk of cancer-specific mortality and all-cause mortality in lung cancer patients [21]. There is also evidence of poor survival in patients with metastatic lung cancer and persistent depression [22]. The results highlight the need for treatment and psycho-oncological care for lung cancer patients. Because cancer patients and their family caregivers are affected by their own and each other’s mental and physical health, it seems essential to provide psycho-oncological care to family members when needed. The aim of a holistic treatment should therefore include the support of the relatives to achieve better care. Therefore, psycho-oncological guidelines also recommend the support of relatives and other informal caregivers [23, 24]. A systematic review on psychological interventions for informal caregivers of lung cancer patients examined 22 studies with interventions classified into four categories, i.e., communication-based, coping skill training, multicomponent, and stress reduction. Most of the interventions with various delivery modes showed improved burden, anxiety, depression, stress, overall quality of life, self-efficacy, and coping skills, although not always statistically significant [25]. They conclude that the caregiver’s burden can be alleviated through support and resource creation in the context of psychosocial interventions. However, heterogeneity in design, methodology, interventions, and sample characteristics was challenging, and further research with more rigorously designed studies and robust analyses to establish an evidence‐based practice is required.

A tool that might be helpful in the psychological support of relatives is the Managing Cancer And Living Meaningfully (CALM) intervention. CALM is a brief, tailored, supportive-expressive psychotherapeutic intervention that intends to treat and prevent depression and end-of-life distress in patients with advanced cancer. It provides a therapeutic relationship and reflective space about four domains: symptom management and communication with health care providers, changes in self and relations with close others, spiritual well-being and the sense of meaning and purpose, and mortality and future-oriented concerns [26]. CALM is feasible and effective in patients with advanced cancer. Patient-identified unique benefits of the intervention are [1] a safe place to process the experience of advanced cancer, [2] permission to talk about death and dying, [3] assistance in managing the illness and navigating the healthcare system, [4] resolution of relational strain, and [5] an opportunity to “be seen as a whole person” within the healthcare system [27]. A controlled study showed the positive effects of CALM on depressive symptoms, coping, and psychological well-being in patients with advanced cancer [28]. However, to our knowledge, no study applied the CALM manual to support relatives. Therefore, we conducted the following study with relatives of patients with lung cancer.

## Objectives

Our study aims to prove the effectiveness of a structured psycho-oncological intervention based on the CALM (CALM intervention) compared to usual care for relatives of lung cancer patients. In a randomized controlled study, we examine the following research questions:

The following are the main research questions:

- Does a CALM intervention for relatives of lung cancer patients (IG) reduce the extent of self-reported anxiety *(primary outcome*) compared to usual care (CG)?
- Does a CALM intervention for relatives of lung cancer patients (IG) reduce self-reported symptoms of depression, distress, and supportive care needs while increasing quality of life (*secondary outcomes*) compared to the CG?

We hypothesize that the CALM intervention for relatives of lung cancer patients reduces the extent of reported anxiety among relatives (*primary hypothesis*). In addition, we expect superior effectiveness of the CALM intervention regarding depression, distress, supportive care needs, and quality of life.

As a *secondary research question*, we explore the impact of psycho-oncological support of relatives on the patients’ mental health outcomes.

- Does the psycho-oncological support of relatives positively affect patients’ levels of depression, anxiety, and distress indirectly?

We expect that a supported and resource-empowered relative has an indirect positive effect on the patient’s well-being.

## Methods/design

### Trial design

**Fig. 1:**
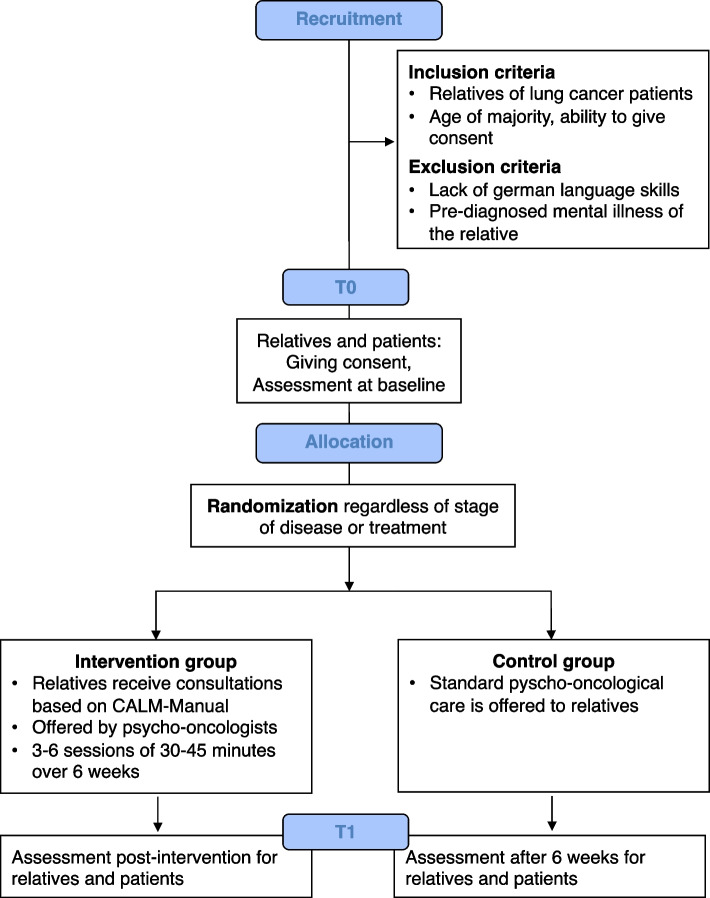
Study protocol diagram of data collection processes

The study is a single-center, prospective, randomized controlled trial (RCT) with two measurement time points. Relatives of lung cancer patients and, thus, the patients themselves (i.e., dyads) are randomly assigned to the CALM intervention group (IG) or the control group (CG) regardless of their disease or treatment stage. The relatives in the IG will receive psycho-oncological support according to the CALM manual, whereas the relatives in the CG will receive usual psycho-oncological treatment (usual care). However, the patients in both study arms will not get a specific intervention. The relatives and the patients complete the assessments at baseline (T0) and after the intervention (IG, T1) or after 6 weeks (CG, T1). Figure 1 shows the study workflow, and Table 1 shows the SPIRIT reporting guideline schedule.

**Table 1:** SPIRIT schedule of enrollment, interventions, and assessments

	**Study period**				
	**Enrollment**	**Baseline**	**Allocation**	**Post-allocation**	
**Time point**	*** − t***_**0**_	***t***_**0**_			***t***_**1**_
**Enrollment**					
Eligibility screen	X				
Informed consent	X				
Randomization			X		
**Interventions**					
Intervention: CALM					
Control: usual care					
**Assessments**					
**Relatives**					
GAD-7		X			X
PHQ-9		X			X
DT		X			X
SNCS		X			X
CQOLC		X			X
Sociodemographic data		X			
**Patients**					
GAD-7		X			X
PHQ-9		X			X
DT		X			X
Sociodemographic data		X			
Medical data		X			

### Study setting and participants

This study is performed at the University Hospital Würzburg, Comprehensive Cancer Center Mainfranken (CCCMF), Germany.

The eligibility criterion for participants is being a relative of a patient with lung cancer (ICD-10: C34). Relatives are defined as spouses, children, parents, other family members, and close friends or caregivers. Relatives and patients are included regardless of the stage of disease, histology, staging, and choice of therapy or treatment. Further criteria are the age of 18 years or older (of the relative and the patient) and the ability to give informed consent. The exclusion criteria are a lack of German language abilities or a pre-diagnosed mental illness of the relative.

### Intervention

#### Intervention group (IG)

The intervention condition is a structured psycho-oncological intervention based on the Managing Cancer And Living Meaningfully (CALM) manual. CALM is an individualized, evidence-based psychotherapeutic intervention that focuses on four domains relevant to the challenges typically experienced by cancer patients:

- Symptom management and communication with healthcare providers
- Changes in self and relations with close others
- Spiritual well-being and a sense of purpose
- Concerns related to the future and mortality [28]

The topics covered in the CALM domains are also relevant for relatives. Adaptations of the intervention therefore refer to the fact that in all domains, the well-being (e.g., physical and psychological symptoms) and the self-perception of the relative (e.g., change in values since the relative’s illness) are explored instead of the well-being and perception of the patient.

The CALM domains should be addressed in a tailored, individualized manner that allows for variation in time spent on each domain and the number of sessions based on the participants’ needs. Therefore, each relative receives three to six counseling sessions, each lasting 45 min. A CALM-trained psychologist delivers the sessions within 6 weeks. Three psychologists of the CCCMF (experienced psycho-oncologists/psychotherapists) were trained to do the CALM intervention at the psycho-oncological department.

#### Control group (CG)

Relatives of the CG receive written information about the offers (e.g., counseling, relaxation groups) of psycho-oncological care at the CCCMF by giving the patient the information flyer of the psycho-oncological department. This offer of psycho-oncological care with single and group interventions for patients and relatives corresponds to usual care. Therefore, psycho-oncological counseling is also possible in the CG but is based on personal needs and active engagement. This needs-based counseling in usual care is not structured according to the CALM approach. Furthermore, it is not standard to schedule as many counseling sessions within 6 weeks. This control condition was chosen for ethical reasons.

No specific psycho-oncological intervention is proactively offered to the lung cancer patients in both study arms. However, they can request the support of the psycho-oncological team of the CCCMF (usual care).

#### Treatment integrity

We document the number of counseling sessions in both study groups. As mentioned, there is no restriction for other (psycho-oncological) treatments or counseling.

### Outcomes and measurements

The primary outcome is anxiety. Secondary outcomes are depression and distress for both relatives and patients, supportive care needs, and quality of life for relatives. All outcomes are assessed using self-report validated questionnaires. In addition, sociodemographic data are collected by self-report and medical data by the medical record. Table 2 presents an overview of outcomes and assessments.

**Table 2:** Outcomes, measures, and assessments

Outcomes	Measures	Items	Assessment			
			**Relatives**		**Patients**	
		***n***	**T0**	**T1**	**T0**	**T1**
Anxiety	Generalized Anxiety Disorder-7 Questionnaire (GAD-7) [29]	7	X	X	X	X
Depression	Patient Health Questionnaire-9 (PHQ-9) [30]	9	X	X	X	X
Distress	Distress Thermometer (DT) [31]	1	X	X	X	X
Unmet needs	Supportive Care Needs Survey (SNCS) [32]	34	X	X	–	–
Quality of life	Caregiver Quality of Life Index-Cancer (CQOLC) [33]	35	X	X	–	–
**Further variables**						
Sociodemographic data	Gender, age, education level, relationship to family member/patient, previous psychological treatment	5	X	–	X	–
Medical data	Date of diagnosis, histological subtype, UICC tumor stage, smoking	4	–	–	X	–
Treatment integrity	Number of counseling sessions	1	–	X	–	X

*T0* baseline, *T1* after intervention/6 weeks, *UICC* Union for International Cancer Control

#### Anxiety

The Generalized Anxiety Disorder-7 questionnaire (GAD-7) is a valid and reliable short instrument for assessing symptoms of a generalized anxiety disorder based on the diagnostic criteria of the DSM IV [29, 30]. Seven items record the frequency of anxiety symptoms in the last two weeks using a 4-point Likert (0 = not at all; 1 = several days; 2 = more than half of the days; 3 = nearly every day). Items are summed up to a score ranging from 0 to 21, with higher values indicating higher anxiety [29]. The GAD-7 is particularly useful for assessing the severity of anxious symptomatic and monitoring changes over time [30].

#### Depression

The Patient Health Questionnaire-9 (PHQ-9) assesses the severity of depressive symptoms in the past 2 weeks [31]. The questionnaire consists of nine items derived from the DSM IV/DSM V diagnostic criteria and has high content validity [32]. The items are scored on a 4-point Likert scale (0 = not at all; 1 = several days; 2 = more than half of the days; 3 = nearly every day). The sum score may range from 0 to 27, with higher values indicating higher depression [31].

#### Distress

The Distress Thermometer is a simple and sensitive screening tool to assess psychosocial stress in oncology patients [33, 34]. It assesses distress within the last week on an 11-point rating scale from 0 (not at all stressed) to 10 (extremely stressed). A cutoff value of 5 or more indicates high distress and need for support [33].

#### Unmet needs

The short form of the Supportive Care Needs Survey (SNCS) [35] measures the nature and extent of the perceived needs of oncological patients with 34 items in 5 dimensions: health care system and information; psychosocial well-being; physical well-being; daily living, patient care, and support; and sexuality. On a 5-point Likert scale, patients indicate whether and to which extent they need support (0 = no need, not applicable; 1 = no need anymore, already have support; 2 = low need; 3 = moderate need; 4 = high need) [36]. A standardized value between 0 and 100 and an adjusted value can be calculated [37].

#### Quality of life

The Caregiver Quality of Life Index-Cancer (CQOLC) is used to assess the quality of life of the relatives of cancer patients. The CQOLC is a valid and reliable questionnaire that measures cancer’s psychosocial effects on relatives using 35 items. Several statements regarding how they have felt in the last few days must be rated on a 5-point Likert scale (0 = not at all; 1 = a little; 2 = somewhat; 3 = quite; 4 = very much) [38]. A higher total score (range 0–140) indicates a better health-related quality of life.

For the primary and secondary outcomes, differences between the IG and CG at T1 will be assessed using aggregated questionnaire scores (mean values).

### Sample size calculation

A recent systematic review of interventions for relatives of cancer patients with partly different tumor entities showed heterogeneous results. Many of the included studies showed nonsignificant improvements in outcomes due to insufficient power [25]. Following previous intervention studies with this patient group, which focus on the support of relatives, an effect size of *d* = 0.4 is set [25]. To demonstrate an effect of *d* = 0.4 in the primary outcome (GAD-7) at a significance level of alpha = 0.05 and beta = 0.2 for comparing two independent samples, *n* = 200 relatives are required (100 per group). Due to heterogeneous results and high effort in recruiting this vulnerable group, we plan an interim analysis with *n* = 88 persons (44 per group; sample size showing a medium effect size *d* = 0.6). The trial will be closed if the effect size is *d* = 0.6 or *d* < 0.4. A member of the statistical consulting of the Institute of Clinical Epidemiology, University of Würzburg, supported sample size calculation.

### Recruitment

Lung cancer patients treated in the University Hospital Würzburg and their relatives are recruited either in the pneumatological outpatient clinic or by contacting the patients or their relatives by phone. Eligible persons are informed about all relevant aspects of the study, i.e., the psycho-oncological intervention for the relatives, the procedure, and assessments. It is also explained that participation in the study is voluntary and can be terminated at any time without giving a reason. In the case the patients and their relatives are interested in the study, they get written study information, a flyer with the offers of the psycho-oncological department of the CCCMF, the baseline set of questionnaires (T0), and an informed consent form (in person or delivered by mail). After written consent is provided and the initial questionnaires (T0) are completed, a psycho-oncologist will contact relatives in the IG to set a date for an initial appointment, whereas CG will not be actively contacted.

### Assignment of interventions

#### Randomization and allocation concealment

All participants, i.e., dyads of relatives and lung cancer patients, will be randomly assigned to IG or CG after consent is given and initial questionnaires (T0) are completed.

A block randomization procedure with an allocation ratio of 1:1 is used. A member of the study center (not involved in the study) created the randomization list with computer-generated random numbers linked to the research codes (i.e., a consecutive list of numbers). The research code is used on all questionnaires and medical data instead of the name (pseudonymization; see confidentiality). The list is stored locked at the study center. Research codes are printed on the first questionnaires (T0). Therefore, assignment based on the sequence of the list is determined by the time of receipt of the interest to participate and, consequently, the distribution of the study information, consent form, and questionnaire. Allocation concealment will be ensured, as the study members responsible for enrollment (i.e., study coordinator) will not release the randomization number until the signed consent form and baseline assessments have been completed.

#### Blinding

Blinding of participants and psycho-oncologists is not possible due to the active counseling intervention. However, psycho-oncologists are not involved in recruitment and data collection. Other care providers are blind to study participation and/or assignment. For organizational reasons, the analysts will not be blinded. The members of the study team will each take on several tasks. They are responsible for enrollment, data collection, management, and statistical analysis.

### Data collection, data management, confidentiality, and retention

Data will be assessed with patient questionnaires (paper–pencil), and medical data will be extracted from the medical record. The baseline set of questionnaires (T0) will be administered in person or delivered by mail. The follow-up questionnaires (T1) will be sent by mail with a prepaid envelope for return. Researchers will check the responses and, if necessary, contact nonresponders via telephone to promote complete follow-up.

All data will be transferred manually to a statistical program and stored on secured servers with regular backup. Double data entry will be processed for 20% of the questionnaires to assess data quality. Furthermore, range checks for data values will be performed. Questionnaires will be stored in locked cabinets. Only the research team will have access to questionnaires and data.

The data are pseudonymized to ensure confidentiality. For this purpose, each study participant is assigned a research code. The code list (including name, date of birth, SAP research number, and contact data) is accessible only to the research member responsible for the assignment and data collection. It is stored locked on an external data carrier that is not accessible to the public or other team members.

The principal investigator will retain all collected data for at least 10 years. The code list will be destroyed at the end of the study.

### Statistical analysis

Statistical analysis will be performed using IBM SPSS Statistics for Windows. Missing data in accordance with missing completely at random (MCAR) or missing at random (MAR) assumptions will be imputed using a multiple imputation procedure. In addition, missing values due to drop-out or data not missing at random (NMAR) will be analyzed by pair-wise deletion.

Descriptive analyses will be performed concerning socio-demographic and medical data.

#### Main research question

The primary and secondary endpoints will be analyzed according to the intention-to-treat principle. Treatment effects (between-group effects) in primary and secondary outcomes will be evaluated using analysis of covariance (ANCOVA) adjusting for baseline values. Statistical significance (*p* < 0.05, two-sided) and effect sizes (*ή*^2^) will be reported for all between-group differences [27]. In addition, within-group effects, including standardized effect sizes (SES) and accompanying 95% confidence intervals (CIs), will be calculated for both study groups.

#### Secondary research question

We will also use ANCOVA adjusting for baseline values to examine the group differences in patients’ mental well-being by psycho-oncological support of their relatives (less than two consultations versus at least two consultations).

#### Interim analysis

Due to heterogeneous results and high effort in recruiting this vulnerable group, we plan an interim analysis on the primary endpoint with *n* = 88 persons (sample size showing a medium effect size *d* = 0.6). The study team will discuss the results and decide on the continuation of the trial. Pre-specified criteria are as follows: The trial will be closed when there is a medium effect of *d* = 0.6 or smaller effects than *d* = 0.4. Recruitment will be continued until the calculated sample size if the effect is about *d* = 0.4.

### Monitoring: harms

The risks of harm to participants in this study are minor. However, dealing with the relative’s illness can also lead to psychological stress. If participants discontinue the intervention, the reasons (e.g., subjective adverse effects on psychological well-being) are documented. A serious adverse event (SAE) for the study is suicidality and suicide, which may or may not be causally related to the intervention or study aspects. SAEs will be documented and immediately reported to the principal investigator.

### Ethics, consent, and permission

The study conformed to the principles of the Declaration of Helsinki. The Ethics Committee of the Medical Faculty of the University of Würzburg approved the study protocol on 17.09.2020 (No. 16/20-me). All changes to the study protocol will be submitted to the Ethics Committee for approval. The planned interim analysis was submitted on 22.11.2022 and was approved on 19.12.2022. Participation in the study is voluntary and based on written informed consent. Eligible participants will be informed about all relevant aspects of the study, in particular, that participation in the study is voluntary, and they can withdraw their consent at any time without incurring any disadvantages.

The study team members will publish the study protocol and study results; there are no publication restrictions. The principal investigator will coordinate authorship according to the research questions and tasks of the study team. No professional writers are planned.

## Discussion

Psycho-oncological care usually focuses on the patients themselves. However, relatives of lung cancer patients often suffer from distress that might affect their mental health and quality of life. Psychosocial interventions can alleviate this burden. However, structured interventions for relatives are not usual care so far. Furthermore, further research on interventions for different types of cancer and well-powered trials are warranted. In this RCT, we evaluate the short-term effectiveness of a CALM intervention compared to usual psycho-oncological care for relatives of lung cancer patients. In addition, the effects on the patients will be explored. The study aims to evaluate individual CALM counseling for relatives. Individual counseling is different from couple/family counseling, and we therefore decided on a clear delineation.

Methodological challenges might arise from the delineation of study conditions. For example, based on ethical concerns, it is also possible for the relatives in the CG to receive psycho-oncological support at the CCCMF or other psychosocial cancer counseling centers (usual care). On the other hand, counseling in the IG varies between 3 and 6 sessions, and relatives can decline conversations at any time. Consequently, it is also interesting to compare relatives who had no or few conversations with relatives who experienced more intensive care, quantifiable, for example, by the number of meetings as an independent variable. Furthermore, the estimation of effect size is challenging due to the heterogeneous results of previous studies. On the other hand, there is a high effort in recruiting this vulnerable group and human resources to deliver the structured intervention. Therefore, we plan an interim analysis to estimate the effects considering resources and meaningfulness.

Limitations are that the study period is restricted, and only short-term effects at the end of the intervention will be evaluated. Therefore, the effects over more extended periods will remain unclear. Further studies will be warranted to examine the longer-term effectiveness of the CALM intervention for relatives.

If the CALM intervention shows positive effects on the psychosocial outcome of the relatives, the CALM intervention should be implemented in routine care. Altogether, study results will contribute to a better understanding of the effectiveness of a psycho-oncological intervention for highly impaired cancer patients and their relatives.

## Trial status

This trial is at protocol version 3.1, dated 20.10.22. Date of first enrollment: 30.09.2020.

## Trial registration data set

See Additional file 1: Table S1.

### Supplementary Information

**Additional file 1:** **Table S1.** Trial registration data.

## Data Availability

Only members of the research team will have access to the final data set. There are no data sharing plans.

## Abbreviations

CALM: Managing Cancer And Living Meaningfully
CG: Control group
CQOLC: Caregiver Quality of Life Index-Cancer
GAD-7: Generalized Anxiety Disorder Scale-7
IG: Intervention group
PHQ-9: Patient Health Questionnaire
RCT: Randomized controlled trial
SNCS: Supportive Care Needs Survey
