# Supplementary material for: Effectiveness of structured psycho-oncological counseling for relatives of lung cancer patients based on the CALM approach—study protocol of a randomized controlled trial
Source: Trials. 2024 Feb 10;25:115. doi: 10.1186/s13063-024-07954-9 (PMC10858519; doi:10.1186/s13063-024-07954-9)
Supplement: Supplementary file 1 — Additional file 1: Table S1. Trial registration data. [file 13063_2024_7954_MOESM1_ESM.docx]

Supplement

Table S1. Trial registration data

| **Data catagory** | **Information** |
| --- | --- |
| Primary registry and trial identifying number | DRKS-ID: DRKS00030077 |
| Date of registration in primary registry | 26 October 2022 |
| Source(s) of monetary or material support | University Hospital Würzburg, Comprehensive Cancer Center Mainfranken; Institutional budged/no external funding |
| Primary sponsor | University Hospital Würzburg, Comprehensive Cancer Center Mainfranken |
| Contact for public queries | Dr. Elisabeth Jentschke (Jentschke_E@ukw.de) |
| Contact for scientific queries | Dr. Elisabeth Jentschke (Jentschke_E@ukw.de) |
| Public and scientivic title | Effectiveness of psycho-oncological care for relatives of lung cancer patients |
| Countries of recruitment | Germany |
| Health condition(s) or problem(s) studied | Lung cancer |
| Intervention(s) | Study group: structured psycho-oncological counseling based on the CALM (Managing Cancer And Living Meaningfully) manual |
|  | Control group: usual care |
| Key inclusion and exclusion criteria | Inclusion criteria: Relatives of patients with lung cancer; male and female; age of 18-years or older |
|  | Exclusion criteria: Lack of German language abilities, mental illness (before cancer diagnosis) |
| Study type | Interventional |
|  | Allocation: randomized |
|  | Primary purpose: supportive care |
| Date of first enrolment | September 2020 |
| Target sample size | 88 |
| Recruitment status | Recruiting |
| Primary outcome(s) | Anxiety (GAD-7) after intervention |
| Key secondary outcomes | Depression, psychosocial distress, supportive care needs, quality of life after intervention |
